# Patient Derived Organoids Confirm That PI3K/AKT Signalling Is an Escape Pathway for Radioresistance and a Target for Therapy in Rectal Cancer

**DOI:** 10.3389/fonc.2022.920444

**Published:** 2022-07-04

**Authors:** Kasun Wanigasooriya, Joao D. Barros-Silva, Louise Tee, Mohammed E. El-asrag, Agata Stodolna, Oliver J. Pickles, Joanne Stockton, Claire Bryer, Rachel Hoare, Celina M. Whalley, Robert Tyler, Toritseju Sillo, Christopher Yau, Tariq Ismail, Andrew D. Beggs

**Affiliations:** ^1^ Institute of Cancer and Genomic Science, College of Medical and Dental Science, University of Birmingham, Birmingham, United Kingdom; ^2^ Department of Surgery, University Hospitals Birmingham National Health Service (NHS) Foundation Trust, Birmingham, United Kingdom

**Keywords:** colorectal cancer, organoid, radiotherapy, PI3K - AKT pathway, mTOR

## Abstract

**Objectives:**

Partial or total resistance to preoperative chemoradiotherapy occurs in more than half of locally advanced rectal cancer patients. Several novel or repurposed drugs have been trialled to improve cancer cell sensitivity to radiotherapy, with limited success. We aimed to understand the mechanisms of resistance to chemoradiotherapy in rectal cancer using patient derived organoid models.

**Design:**

To understand the mechanisms underlying this resistance, we compared the pre-treatment transcriptomes of patient-derived organoids (PDO) with measured radiotherapy sensitivity to identify biological pathways involved in radiation resistance coupled with single cell sequencing, genome wide CRISPR-Cas9 and targeted drug screens.

**Results:**

RNA sequencing enrichment analysis revealed upregulation of PI3K/AKT/mTOR and epithelial mesenchymal transition pathway genes in radioresistant PDOs. Single-cell sequencing of pre & post-irradiation PDOs showed mTORC1 and PI3K/AKT upregulation, which was confirmed by a genome-wide CRSIPR-Cas9 knockout screen using irradiated colorectal cancer (CRC) cell lines. We then tested the efficiency of dual PI3K/mTOR inhibitors in improving cancer cell sensitivity to radiotherapy. After irradiation, significant AKT phosphorylation was detected (*p*=0.027) which was abrogated with dual PI3K/mTOR inhibitors and lead to significant radiosensitisation of the HCT116 cell line and radiation resistant PDO lines.

**Conclusions:**

The PI3K/AKT/mTOR pathway upregulation contributes to radioresistance and its targeted pharmacological inhibition leads to significant radiosensitisation in CRC organoids, making it a potential target for clinical trials.

## Highlights

### What Is Already Known on This Topic?

- Neo-adjuvant chemoradiotherapy treatment in rectal cancer provides patient benefit, and good response to this treatment is associated with better survival
- The molecular mechanisms of this are currently unknown but have been hypothesised to be associated with KRAS mutations or mTOR signalling dysfunction

### What This Study Adds

- Multiple orthogonal methods demonstrate that upregulation of mTOR/AKT/PI3K signalling is critical in causing radioresistance in rectal cancer
- This effect can be overcome in a variety of genetic backgrounds in organoids of rectal cancer using dual AKT/mTOR inhibitors such as dactosilib or apitolisib

### How Might This Study Affect Research Practice or Policy?

- Dual AKT/mTOR inhibitors should be prioritised for a neoadjuvant therapy trial to increase response rates in rectal cancer.

## Introduction

Neoadjuvant chemoradiotherapy (CRT) followed by surgical resection is the standard of care for patients with locally advanced rectal cancer (LARC) (1). It downstages disease, increases rates of sphincter sparing surgery and reduces the risk of local recurrence (2, 3). Approximately 10-30% of patients receiving CRT demonstrate a complete pathological response, where no residual tumour cells are seen on histopathological assessment of post-resection surgical specimens (4). Conversely, around a third of patients demonstrate no response to CRT (5). Despite clinicopathological features (6), the tumour microenvironment (7), microbiome (8),and genomic markers (9) being linked to complete response, a definitive molecular mechanism underlying CRT resistance in rectal cancer remains unclear. Understanding the pathways that drive CRT resistance is essential to developing tools to predict patient response and improve treatment response (9).

The impact of intra- and inter-patient tumour heterogeneity in acquired resistance to therapies is now widely acknowledged. Patient-derived organoids (PDO) can be used as preclinical models that resemble the genomic heterogeneity observed in patients, maintain cellular heterogeneity and genetic stability after multiple passages and respond similarly to treatment both *in vivo* and *in vitro* (10). PDOs have been developed from primary rectal cancer biopsies and surgical resection specimens recapitulating the genetic diversity and treatment response within rectal cancers (11, 12). Ganesh et al. demonstrated that rectal cancer organoids mirror clinical responses of individual patients to chemoradiotherapy (11). Yao et al. observed a broad range of intrinsic PDO responses to conventional chemoradiation (12). These studies suggest PDOs can serve as tumour avatars which predict rectal cancer response to various treatments. We have previously shown tumour hypermutation, dysregulation of PI3K/MTOR signalling and high neoantigen load correlate with response to therapy (13).

In the present study we utilised PDO models to investigate the mechanisms of resistance to radiotherapy and test the hypothesis that these pathways may be druggable thus potentially improving radiotherapy response in rectal cancer. We aimed to do this by comparing the transcriptome of radiosensitive and radioresistant PDOs at the bulk and single cell level, and by carrying out genome-wide clustered regularly interspaced short palindromic repeats (CRISPR)–associated nuclease 9 (Cas9) knockout screens (under radiation selection pressure) of CRC cell lines to cross- validate findings. Finally, we aimed to integrate our analysis of the organoid models to select agents that may increase radiosensitivity compared to the current standard radiosensitising agent (5FU) to evaluate how they affected response to radiation.

## Results

### Patient-Derived Organoids (PDO) Characterization

**Figure 1:**
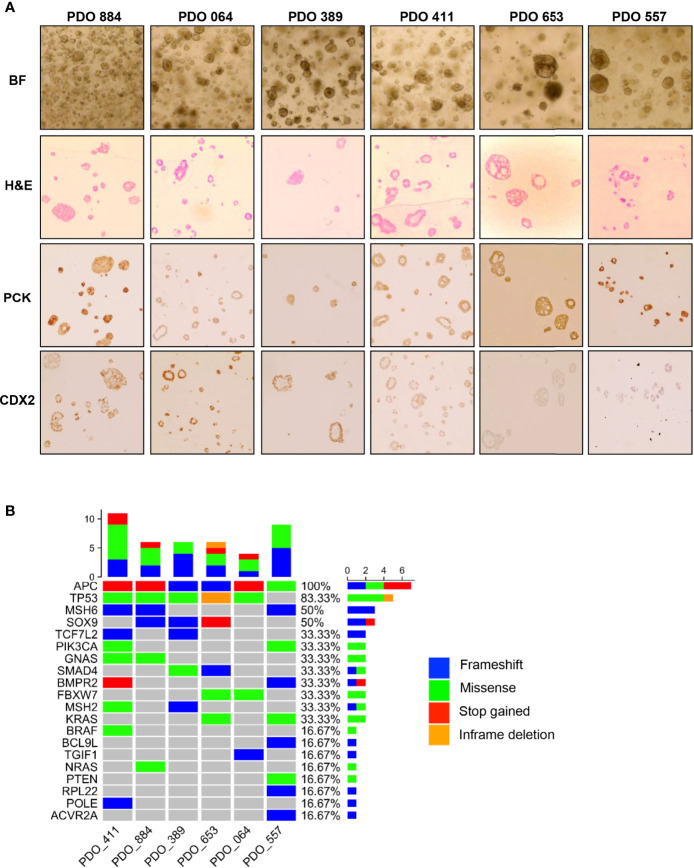
Characterization of the patient-derived organoids used in this study. **(A)** Bright-field microscopy (BF) imaging and H&E staining (H&E) of cultured PDOs; Immunohistochemistry staining of pan-Cytokeratin (PCK) and caudal-type homeobox 2 (CDX2) to compare staining pattern with matching primary tumours. **(B)** Heatmap illustrating the mutational landscape of PDO lines obtained through targeted DNA-sequencing using a panel of 30 known CRC genes (see *Methods* for detailed list).

**Figure 2:**
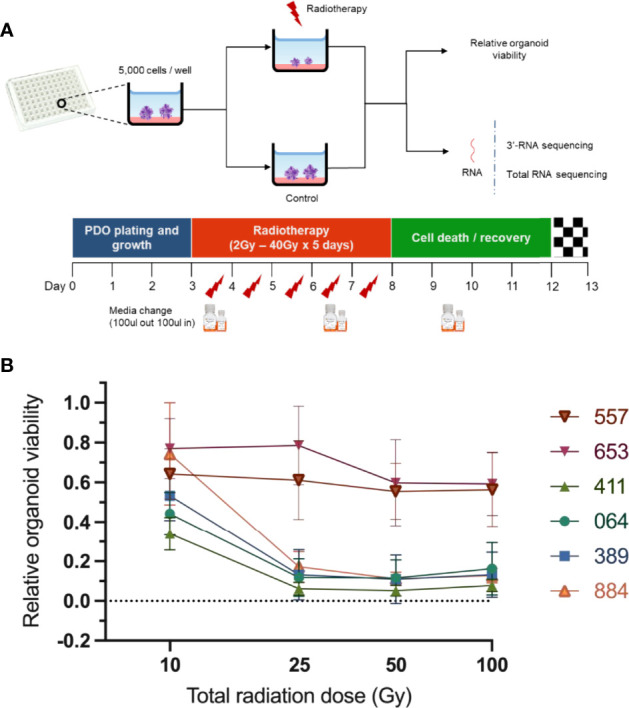
Assessment of PDO lines response to radiotherapy. **(A)** Scheme illustrating experimental design. The PDO lines were cultured in 96-well plates (5000 organoid cells/well) and treated with different radiotherapy doses ranging from 2Gy x 5 days to 20Gy x 5 days. A chemiluminescence-based assay (CellTiter-Glo^®^ 3D) was then used to assess relative PDO. **(B)** Radiation response curves of PDO lines from three independent experiments (error bars represent standard deviation).

Initially we derived over thirty organoid models of rectal cancer, choosing six organoid models to provide depth of examination at the single cell and bulk level, based on their radioresistance. Immunostaining revealed pan-Cytokeratin (to verify epithelial origin) expression on the six PDO lines, matching the primary tumours’ expression (**Figure 1A**). Two PDO lines (653 and 557) showed no staining for the intestinal-specific transcription factor CDX2, matching their primary tumours (**Figure 1A**; **Supplementary Figure 1**). CDX2 is an intestinal specific transcription factor and approximately 20% of CRC do not express CDX2 (14). Targeted DNA sequencing of PDO lines revealed mutations in 20 of the 30 genes included in a colorectal cancer specific driver panel, with *APC* being mutated in all six PDO lines (**Figure 1B**; **Supplementary Table 2**). To evaluate PDO sensitivity to radiotherapy, each line was irradiated with daily fractions – ranging from 2Gy to 10Gy – over 5 days, after which cell viability was assessed (**Figure 2A**). Relative organoid cell viability decreased by 90% in four PDO lines (884, 064, 389 and 411) when treated with a total of 25Gy or higher, whereas for lines 653 and 557 there was a 40% reduction (**Figure 2B**). The former group was deemed radiosensitive and the latter radioresistant. Targeted DNA sequencing revealed *KRAS* mutations (*G12D *and* G13R)* in the two radioresistant PDO lines, with line 653 additionally harbouring a *TP53* deletion *(P191del)* and a missense *FBXW7* mutation *(R465H)*. The 557 PDO line contained a *MSH6* frameshift mutation *(F1104fs)*, and the parent tumour also displayed deficient mismatch repair (dMMR) phenotype on immunohistochemistry. This line also harboured a pathological missense *PIK3CA* mutation (*R88Q)*.

### Bulk RNAseq of Radiosensitive vs. Radioresistance Organoid Lines Demonstrate PI3K/AKT Signalling as Being Significantly Enriched in Organoid Lines

**Figure 3:**
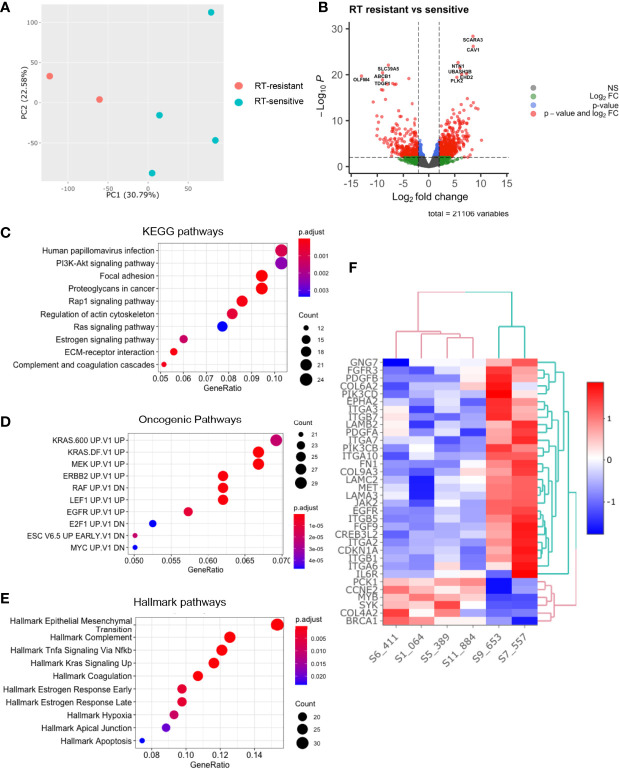
Comparative analysis of baseline transcriptomes of radiation-sensitive and radiation-resistant PDO lines. **(A)** Principal component analysis; **(B)** Volcano plot of upregulated and downregulated genes in radioresistant PDOs; Gene set enrichment analysis using **(C)** KEGG pathways, **(D)** Oncogenic Pathways and **(E)** Hallmarks pathways gene sets. **(F)** Heatmap of upregulated PI3K/AKT/mTOR pathway genes in radioresistant PDOs. (PC=principal component, FC=fold change).

Principal component analysis of the pre-treatment PDO transcriptomes grouped the samples two main clusters (**Figure 3A**) matching the radiation-response status we defined experimentally (**Figure 2A**), suggesting distinctive radiosensitivity gene expression profiles. The transcriptomes of the two radioresistant PDO lines were more closely clustered compared to the radiosensitive PDO lines, identified using our earlier experiments (**Figure 3A**). In total, we observed 9659 differentially expressed genes (fold change [FC] >= 1.5) between the radioresistant and radiosensitive PDO lines (False discovery rate [FDR]< 0.05). Of these, 7,294 were upregulated in radioresistant PDO lines and the remainder were upregulated in the radiosensitive lines. The top five significantly upregulated genes in the radioresistant PDO lines included *SCARA3, CAV1, NTN1, UBASH3B* and *PLK2*, whereas *OLFM4, SLC39A5, ABCB1*, *TDGF1*, and *CYP2B6* were the top five differentially upregulated genes in a radiosensitive PDO lines (**Figure 3B**). To further characterize the transcriptional differences, we performed pathway enrichment analyses using the Hallmark, KEGG and Oncogenic Signatures gene-set collection of the Molecular Signatures Database (MSigDB V7.4). KEGG enrichment analysis revealed several the Human papillomavirus infection pathway genes (24 of 233, *p*=0.001) and the PI3K/AKT signalling pathway genes (24 of 233, *p*=0.002) upregulated in the radioresistant PDOs (**Figure 3C**). The upregulated genes of the latter are represented in **Figure 3F**. Enrichment analysis using the MSigDB Oncogenic Signature gene set revealed that the upregulated genes in radioresistant PDO lines match a set of genes upregulated in *KRAS* mutant cells (**Figure 3D**), and Hallmark pathway analyses also revealed upregulation of Epithelial Mesenchymal Transition related genes (**Figures 3D, E**).

### Single Cell RNAseq & Genome Wide CRISPR Screens Confirm the Significance of PI3K/AKT/mTOR Pathway in Radiation Response

**Figure 4:**
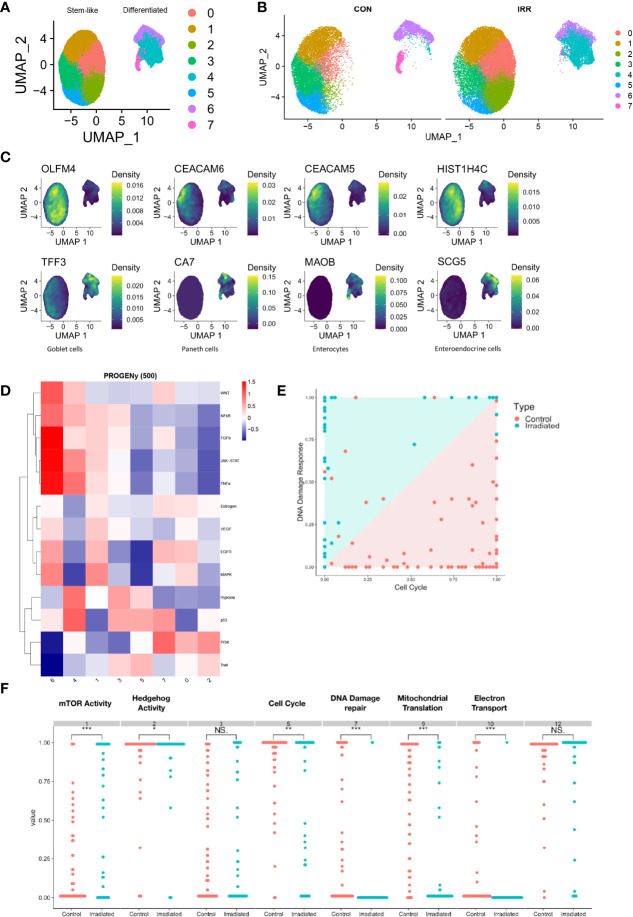
Single-cell RNA sequencing of irradiated PDOs shows cell type specific response to radiation and increase in mTOR activity in radiated cells. UMAP dimensional reduction of single-cell sequencing of irradiated (IRR) and control (CON) radiation sensitive PDOs displayed merged **(A)** and separately **(B)**. **(C)** Markers of stem-like and differentiated clusters. **(D)** Heatmap illustrating pathway responsive genes (PROGENy) for each cell cluster. **(E, F)** Scalable Bayesian Boolean Tensor Factorisation (SBBTF) and pathway analysis showing enrichment of mTOR activity on irradiated cells. *p < 0.05, **p < 0.01, ***p < 0.001, NS, Not significant.

To understand the role of heterogeneity in response to radiation therapy, single-cell RNA sequencing was performed on three paired irradiated and control PDOs. A total of 51,000 cells with 24,011 gene features were identified, which on dimensionality reduction formed two main cell clusters (**Figure 4A**). When the cells were separated by irradiated and non-irradiated groups, a large expansion in the cluster expressing colonic stem cells was identified showing variability when exposed to radiation (**Figure 4B**). The first consisted of *CEACAM5 and CEACAM6* expressing tumour cells, colonic stem cells with high expression of *OLFM4* and *HIST1H4C* (**Figure 4C**). The second cluster consisted of differentiated colon cells with expression of markers of goblet cells, Paneth cells, enterocytes and enteroendocrine cells (**Figure 4D**). This cluster also expressed elevated levels of the BMP ligands BMP2, BMP4 and BMP7 (supplementary data). Pathway analysis by Seurat and PROGENy did not demonstrate any consistently dysregulated pathways (**Figure 4D**). Therefore, dimensionality reduction using Scalable Bayesian Boolean Tensor Factorisation (SBBTF) and pathway analysis was carried out, which showed significant upregulation of genes participating in the mTOR, DNA damage repair, mitochondrial translation and electron transport pathways in irradiated organoids, and significant downregulation of Hedgehog signalling and the cell cycle (**Figures 4E, F**).

**Figure 5:**
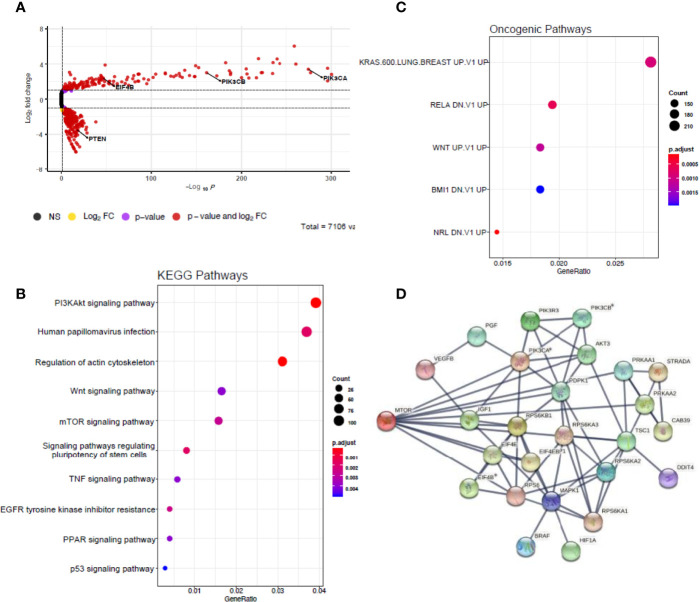
Genome-wide CRISPR screens identify mTOR signalling up-regulates pathway genes. **(A)** Volcano plot depicting significantly differentially expressed genes (p < 0.05 and |fold change| > 1) in red. Model-based analysis of genome-wide CRISPR-Cas9 knockout on libraries A and B detected twenty-six expressed genes from mTOR signalling pathway gene list based on molecular signatures database (MSigDB) v7.4. **(B)** Dot plot showing significantly over-represented KEGG pathway terms that were defined from MSigDB v7.4 using the 3,207 DEGs with p < 0.05. **(C)** Dot plot showing significantly over-represented oncogenic pathway terms that were defined from MSigDB v7.4 using the 3,207 DEGs with p < 0.05. **(D)** Network diagram representing the mTOR signalling events mediated by PIK3CA, PIK3CB and EIF4B using STRING 2 weighted correlation network analysis (WGCNA). Asterisks represent statistically significant levels of gene differences between control and irradiated samples (p< 0.05) calculated using Student’s t test. NS, non-significant; FC, fold change.

To validate this result, a genome-wide CRISPR-Cas9 knockout screen was performed on the C80 (*APC*mt, *KRASmt*, *TP53*mt) and HT55 (APCmt, KRASwt, TP53mt) CRC cell lines. These lines were chosen as one had a KRAS mutation and one did not, given the effects seen by KRAS in the bulk RNA sequencing data. The sgRNA libraries used target 19,050 genes using 123,411 sgRNAs. Differential sgRNA data were processed using MaGECKFlute identifying positively selected genes under the selection pressures of 5 Gy/day irradiation over 5 days (**Figure 5**). The volcano plot in **Figure 5A** depicts significantly differentially expressed genes (*p*<0.05 and FC> 1) in viable cells following irradiation. In total, there were twenty-six genes that showed expression based on the mTOR signalling pathway gene list from the MSigDB. *PIK3CA*, *PIK3CB* and *EIF4B* were differentially upregulated in irradiated samples compared to control samples, and PTEN was downregulated in the irradiated cells (**Figure 5A**). Enrichment analysis using KEGG gene sets of the MSigDB revealed upregulated genes matched the PI3K/AKT/mTOR pathway gene set (**Figure 5B**). The upregulated genes also matched several pathways of the Oncogenic Signatures gene set collection (**Figure 5C**) including KRAS and WNT signalling. Module eigengenes were calculated and hierarchically clustered to merge very closely correlated modules together, reducing the total number of modules. A total of twelve modules (groups of co-expressed genes) were detected by hierarchical clustering and subsequent module merging using eigengenes. mTOR signalling module were detected and visualized using STRING 4 (**Figure 5D**). Negative selection was observed in the entire PI3K/AKT/mTOR pathway, as well as the RAS/RAF/MEK/ERK signalling pathways, suggesting these pathways were involved with the cellular response to radiotherapy. We also observed that the differently expressed pathways in the CRISPR-Cas9 and scRNA-seq dataset overlapped.

### Dual PI3K/mTOR Inhibitors Improve CRC Cell Line Response to Radiation, However Single Agent mTOR Inhibition Does Not

**Figure 6:**
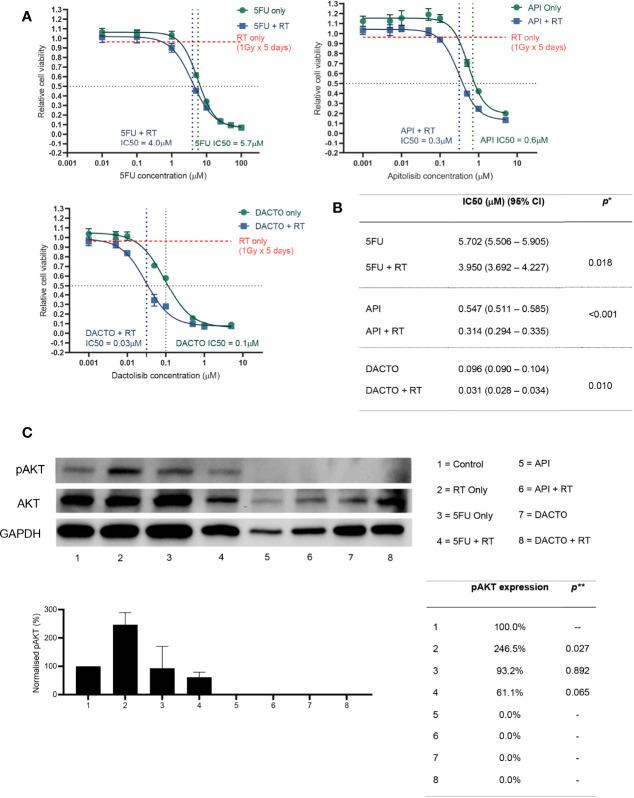
Radiosensitizing HCT116 and its AKT phosphorylation status following drug and/or radiotherapy treatment.**(A)** HCT116 was treated with eight different concentrations of 5-FU, Apitolisib or Dactolisib, with or without radiotherapy (1Gy fractions over 5 days). End-point cell viability was assessed using CellTiter-Glo 2.0 (Promega, USA) and chemiluminescence. The data is presented normalised to control (containing 0.1% DMSO) versus log transformed drug dose concentrations (μM). **(B)** Half maximal inhibitory concentrations (IC50) for each of the drugs with or without radiotherapy were obtained using Prism (Graphpad, USA). A statistically significant difference in radiosensitisation across all three drugs (radiotherapy effect; Two-way ANOVA test*, p < 0.0001) and between the different drug doses (drug effect: p < 0.0001*). **(C)** Results from western blot analysis of protein extracted from HCT116 following a 72-hour incubation with or without the three drugs (5FU-5μM; Apitolisib-100nM, Dactolisib-100nM), and with or without a single 5Gy dose of radiotherapy. Image densitometry was performed and relative pAKT expression has been demonstrated normalised to control (containing 0.1% DMSO). Protein was extracted two-hours after irradiation. Irradiation alone led to a significant increase in AKT phosphorylation at two-hours (p=0.027; two-tailed T-test**). Treatment with Dactolisib and Apitolisib with or without radiotherapy led to complete inhibition of AKT phosphorylation across three independent repeat experiments. API, Apitolisib; DACTO, Dactolisib; RT, radiotherapy; CI, confidence interval; pAKT, phosphorylated AKT; DMSO, Dimethyl sulfoxide.

Following the findings of PIK3/AKT axis up-regulation in radioresistant PDO, we initially tested both Sirolimus and Everolimus (mTORC1 inhibitors) effect on the HCT116 cell line combined with radiotherapy, finding no measurable difference in cell viability (data not shown). We hypothesised that mTORC inhibition was not effective because of alternate mechanisms for pAKT activation in the signalling pathway. Initially we tried sensitising organoids with the pan-AKT inhibitor MK-2206 and the mTORC1/2 inhibitor vistusertib (AZD2014) with no significant effect on radiosensitisation (data not shown). We then tested the effect of the PI3K/mTOR dual inhibitors (Apitolisib and Dactolisib) combined with radiotherapy (1Gy daily fractions for 5 days) on survival of CRC cells (HCT116) and how these compared with 5FU treatment. Radiotherapy as a standalone treatment only reduced cell line viability by less than 10% of baseline. The IC50 for HCT116 treated with either Apitolisib or Dactolisib were 0.6μM, 0.1μM without radiotherapy, and 0.3μM, 0.03μM with radiotherapy, demonstrating that both apitolosib and dactosilib caused significant radiosensitisation. Previous studies assessed maximum safe plasma concentrations (Cmax) of Apitolisib to be 0.320μM to 0.380μM upon 30mg or 40mg of drug administered to patients (15). Similarly, Cmax of 0.100μM, 0.220μM and 0.520μM were reported following the administration of 200mg, 400mg or 800mg of oral Dactolisib to patients (16). To confirm target specificity and knockdown, AKT phosphorylation levels assessed 2 hours post-radiation were increased significantly in HCT116 cells two hours after single 5Gy radiotherapy fraction (*p*=0.027; **Figure 6C**) and concomitant treatment with 5FU (5µM) did not significantly suppress AKT phosphorylation (*p*>0.05). However, following treatment with of dual PI3K/mTOR inhibitors (Dactolisib or Apitolisib) there was complete suppression of AKT phosphorylation. AKT phosphorylation remained completely suppressed following administration of radiotherapy to HCT116 cells in the presence of dual PI3K/mTOR inhibitors.

### Dual PI3K/mTOR Inhibitors Enhance Radioresistant PDO Response to Radiation

**Figure 7:**
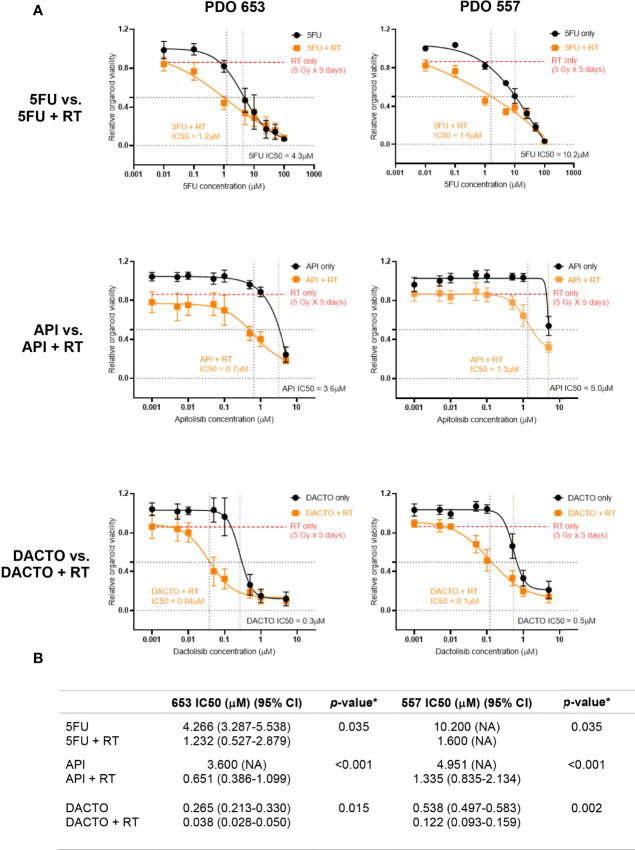
Radiotherapy resistant PDO lines 557 and 653 treated with 5FU, Apitolisib and Dactolisib with or without radiotherapy. **(A)** PDO lines were plated at an estimated 5000 cells/well and treated with was treated with eight different concentrations of 5FU, Apitolisib (API) or Dactolisib (DACTO), with or without radiotherapy (5Gy fractions over 5 days). End-point cell viability was assessed using CellTiter-Glo^®^ 3D (Promega, USA) and chemiluminescence. The data is presented normalised to control (containing 0.1% DMSO). The x-axis represents log transformed drug dose concentrations (μM). **(B)** Half maximal inhibitory concentrations (IC50) for each of the drugs with or without radiotherapy were obtained using Prism (Graphpad, USA). A statistically significant difference in radiosensitisation across all three drugs (radiotherapy effect; Two-way ANOVA test*, p < 0.0001) and between the different drug doses (drug effect: p < 0.0001*).

We then investigated the radiosensitising potential of the dual PI3K/mTOR inhibitors on our radioresistant and radiosensitive organoid model systems. As expected, Dactolisib or Apitolisib used as a single agent was not effective at reducing cancer cell viability. The IC50 following Apitolisib-only treatment for lines 557 and 653 were 5.0μM and 3.6μM; combined with radiotherapy the IC50 dropped to 1.3μM and 0.7μM. The IC50 following Dactolisib-only treatment for lines 557 and 653 were 0.5μM and 0.3μM; combined with radiotherapy the IC50 was 0.1μM and 0.04μM (**Figure 7**).

## Discussion

LARC patients who achieve pathological response following CRT demonstrate improved disease-free and overall survival compared to non-responders or partial responders (17). Furthermore, pre-operative detection of a complete clinical response following CRT, enables the use of “watch and wait” strategies in the management of LARC patients (18), potentially avoiding the morbidity of major surgery. The role of CRT in the treatment of early rectal cancer is also being investigated (19). Therefore, understanding the biological mechanisms underlying radioresistance in LARC will be important to improve pathological and clinical response rates. To address this, we used PDOs given their proven ability to function as tumour avatars that recapitulate the parent tumour genome and mimic clinical response to therapy.

We found *KRAS* mutations (*G12D* and *G13R*) in the radioresistant PDOs, and analysis of their transcriptome showed enrichment of KRAS downstream target genes (**Figure 3C**). *KRAS* mutation status is a predictor of poor response to anti-EGFR antibody therapy in metastatic CRC, and its correlation with rectal cancer response to CRT has been widely studied although a consensus was not reached (20). A systematic review and meta-analysis of 696 patients concluded that *KRAS* status was not predictive of tumour downstaging following CRT in LARC patients (21).However we hypothesise that significant molecular heterogeneity exists within these studied tumours, and that the presence of a KRAS mutation at a level undetectable by conventional sequencing, may lead to the domination of this KRAS mutation clone when irradiated.

After evaluating PDOs response to radiation, we aimed to identify novel genes and pathways that promote radiation resistance by comparing the pre-treatment transcriptomes of radiation-sensitive and radiation-resistant PDOs. The most significantly upregulated genes found in radioresistant PDOs (**Figure 3B**) have known functions of modulating cell response to radiation or are associated with cancer progression. The genes *CAV1* and *SCARA3* have been previously described to be involved in ionizing radiation response or resistance. *CAV1* encodes an integral membrane protein that has been found over-expressed or mutated in solid human tumours and has been shown to be associated with radioresistance (22–24). Other significantly over-expressed genes in radioresistant PDOs – namely *PLK2*, *UBASH3B*, and *NTN1* – have been associated with cancer progression. Ou et al. found that colorectal tumours expressing high levels of Polo-like kinase 2 *(PLK2)* display lower disease-free survival (25). The authors further showed *PLK2* promotes tumour growth and inhibits apoptosis of CRC cells *in vitro* and *in vivo* by binding to *FBXW7* and subsequently promoting its degradation, which in turn leads to the stabilization of Cyclin E, similar to our previous finding that FBXW7 mutations seemed to be associated with radioresistance (13). High expression of netrin1 (NTN1) – a laminin-related secreted protein – is found in at the base of colon crypts, where the intestinal stem cells are located (26) and triggers anti-apoptotic signalling on cells presenting the dependence receptors DCC and UNC5H (27).

Principal component analysis of pre-treatment PDO transcriptomes showed general sample diversity reflecting patient inter-tumour variability and genetic heterogeneity as seen in our single cell transcriptomic data. Notwithstanding, it grouped the PDOs in two clusters according to radiosensitivity (**Figure 3A**).

Radioresistant PDOs displayed upregulation of the epithelial-mesenchymal transition (EMT) pathway and the PI3K/AKT/mTOR pathway (**Figure 3E**), both of which have been associated with radioresistance in rectal cancer (28–30). The role of EMT is well established in cancer metastasis and therapy resistance. This could suggest rectal tumours enriched with cancer cells in a mesenchymal state (e.g., CMS4) are more radioresistant. A close association between PI3K/AKT/mTOR pathway activation and EMT pathway protein expression leading to radioresistance has been previously described (31, 32). The results from our PDO single cell sequencing revealed upregulated PI3K/AKT/mTOR pathway and DNA damage repair genes following irradiation. Genome wide CRISPR-Cas9 knockout screen demonstrated that this pathway was crucial for the survival of irradiated CRC cells. Radiation-resistant tumours are known for the ability to evade apoptosis, enhanced DNA double-strand breaks repair, resistance to hypoxia, poor inflammation, and abundance of tumour stem cell populations. The PI3K/AKT/mTOR pathway regulates several of these functions, and has been implicated in CRT resistance in various cancers (33, 34) and Mutations affecting this pathway genes are frequently detected in cancer. Both of our radioresistant organoids had *KRAS* mutations however one of our sensitive organoids (884) had an *NRAS* mutation. Multiple studies (20, 35) have demonstrated no link between RAS mutations and resistance to radiotherapy, which our findings would seem to support.

Several pharmacological inhibitors targeting the PI3K/AKT/mTOR pathway have been tested for cancer treatment (36) and the radiosensitising potential of these inhibitors is currently being assessed in several tumour types (37). In CRC, studies using cell lines and cell line-derived xenografts have shown that PI3K/mTOR inhibitors Dactolisib and PI-103 improve the efficacy of ionizing radiation (37). As long-term administration of PI-103 before irradiation may cause reactivation of PI3K and MAPK pro-survival pathways, it was excluded from our experiments. This represents a potential source of bias; however, given our results we believe this is not the case. Early clinical trials evaluating the radiosensitising effects of selective mTOR inhibitors such as Everolimus, Rapamycin or Sirolimus failed to increase pathological response rates (37). Recent preclinical evidence proposing dual pathway component inhibitors are effective radiosensitisers and previous use in phase Ib and phase II clinical trials supported our selection of Apitolisib and Dactolisib.

In our assays, Dactolisib showed radiosensitising effects in HCT116 and radioresistant PDOs, with an IC50 lower than the previously published C_max_ in humans. Conversely, the IC50 for Apitolisib-treated radioresistant PDOs exceeded the drugs Cmax in humans, rendering not useful for clinical practice. The different pharmacological properties of the two drugs may explain the differences observed between Apitolisib and Dactolisib response in PDOs. Our results also demonstrated AKT phosphorylation when HCT116 was treated with clinically comparative doses of radiotherapy (**Figure 6C**). We observed complete p-AKT suppression in Apitolisib and Dactolisib-treated cells (**Figure 6**) suggesting that these drugs caused radiosensitivity by a mTOR/AKT/PI3K associated mechanism.

We acknowledge the limited sample size in this study, however the effect of dual mTOR/AKT inhibition was consistent across all rectal cancer organoid models, suggesting that profiling more organoid models would have been redundant. Additionally, our study is the first to demonstrate this in cancer organoid models which have higher fidelity than cell lines and appears to overcome treatment resistance in pre-treated cancers. However, it is conceivable that rare molecular subtypes of rectal cancer may exist that would be resistant to dual mTOR/AKT inhibition, and this could only be determined by screening on a very large bank of PDO. Subsequent studies encompassing larger and more homogeneous cohorts are warranted to further evaluate the effects of radiotherapy and pathway blocking drugs on PI3K/AKT/mTOR pathway downstream effectors and rarer subtypes of rectal cancer.

In summary, this study utilised patient-derived primary cell culture models to investigate the biological mechanisms driving radiation resistance in rectal cancer. We have shown that one of the mechanisms by which rectal cancer attains radioresistance is by upregulation of PI3K/AKT/mTOR. By pharmacological inhibition, we have demonstrated that dual PI3K/mTOR inhibitors could improve radiotherapy efficiency on radioresistant PDOs, warranting further studies to validate the use of these drugs in the clinical setting.

## Methods

### Patient Samples

Patients undergoing surgery for primary colonic or rectal adenocarcinoma were recruited from Queen Elizabeth Hospital Birmingham, UK. Fresh tumour tissue specimens sampled from surgical resection by pathologists were used for tumour organoid derivation. Surgical resection specimens were chosen over tumour biopsies as we wished to establish organoids that reflected tumour heterogeneity and were more likely to be successful as biopsy of the surface tumour does not accurately reflect the full tumour environment. Tumour regression grading in the resection specimen was scored *via* the Mandard System and used to select organoids. A total of 6 PDOs successfully passed the derivation and propagation stage (**Table 1**). Four were treatment naïve (064, 389, 411, 557), whereas 884 received short-course chemoradiotherapy and 653 received long-course chemoradiotherapy. We chose to include two post-treatment organoids in this study as we wished to understand whether the intrinsic resistance seen in these tumours (due to pre-treatment) could be overcome in the same manner as treatment naïve tumours. We have previously demonstrated by analysis of pre-treatment biopsies (13) that mTOR/PI3K/AKT signalling is the primary upregulated mechanism seen to cause treatment resistance and therefore we justifiably expected it to be similar in this study. Anonymised clinicopathological data were obtained from the local Human Biomaterials Resource Centre in Birmingham.

**Table 1:** Clinicopathological characteristics of the parent tumours from which the six PDO lines were derived.

ID	Age	Sex	Diagnosis	Nx	TRS	TNM	MMR	*KRAS*	*BRAF*
884	75	M	Adenocarcinoma	SCRT	TRS-3	pT3, N2a, M0	No loss	wt (N-RAS-mut)	wt
064	79	M	Adenocarcinoma	NA	NA	pT2, N1, M0	No loss	wt	wt
389	82	M	Adenocarcinoma	NA	NA	pT4a, N2a, M0	No loss	wt	wt
411	74	F	Adenocarcinoma	NA	NA	pT3, N2b, M0	No loss	wt	mut
653	55	F	Adenocarcinoma	NCRT	TRS-2	pT2, N0, M0	No loss	mut	wt
557	76	F	Adenocarcinoma	NA	NA	pT3, N0, M0	Loss of MLH1, PMS2 and MHS6	mut	V600E

M, male; F, female; Nx, neoadjuvant treatment; NCRT, neoadjuvant chemoradiotherapy; SCRT, short course radiotherapy; TRS, Tumour regression score; wt, wild type; mut, mutant; NA, not applicable.

### Organoid and Cell Culture

Tumour resection specimens were processed into single-cell suspension by mechanical and enzymatic digestion, mixed with Matrigel^®^ (Corning, USA) and incubated in 50µl droplets on 24-well plates, as previously described by Sato et al. (38). Organoid culture media (OCM) Human Intesticult™ (Stemcell Technologies Inc, Canada) containing 0.2% Primocin™ (*In vivo*gen, USA) and passaged once confluent. Isogenic *KRAS* HCT116 (Horizon Discovery, Cambridge, UK) and C80 (#12022904-1VL, Sigma Aldrich, USA) CRC cell lines were cultured in McCoy’s 5a Medium (Gibco, USA) with 10% fetal bovine serum (FBS, Gibco, USA), Iscove’s modified Dulbecco’s media (Gibco. USA) containing 10% FBS, 2mM L-Glutamine (Gibco, USA) and Eagles modified essential medium (Gibco, USA) with 2mM L-Glutamine containing 1% non-essential amino acids (Sigma-Aldrich, USA), respectively, and one hundred units/ml penicillin and 100 μg/ml streptomycin (Gibco, USA). Cells were passaged when sub-confluent at a split ratio of 1 in 3. All cultures were tested for mycoplasma using EZ-PCR Mycoplasma Test Kit (Biological Industries, Israel).

### Organoid Irradiation Experiments

Suspensions of 5,000 cells from each PDO line were added to Matrigel-coated wells of 96-well plates. Different doses of radiation (2, 5, 10, 20 and 40 Gy) were delivered over five consecutive days using a CellRad irradiator (Faxitron Bioptics, AZ, USA). The irradiation protocol was chosen to closely mimic the short course radiotherapy protocol given in rectal cancer, where 5 days of 5Gy/day is delivered to the rectum. A minimum of four replicates were used per experimental condition. PDOs were incubated for additional 5 days to allow cell recovery to assess cell viability within the range of sensitivity of the cell-viability protocol. Cell viability was assessed by luminescence following the CellTiter-Glo^®^ 3D Cell Viability Assay protocol (Promega, USA). Data were normalized to the PDO wells treated with 0 Gy as 100% viability to calculate relative cell viability of the irradiated wells.

### Immunohistochemistry

PDOs were released from Matrigel^®^ drops, fixed in 4% paraformaldehyde and cast in 2% agarose pellets. These were then embedded in formalin-fixed paraffin embedded (FFPE) blocks using standard protocols. Slides with 5µm organoid sections were stained for haematoxylin and eosin (Sigma Aldrich, USA) for morphology assessment. FFPE sections of all PDOs and their respective tumour of origin were stained with antibodies targeting pan-Cytokeratin (ab27988, Abcam, UK) and CDX2 (9272S, Abcam, UK) following standard protocols. The primary antibody dilution was 1/500 and 1/100, respectively, and both were incubated for 1 hour.

### Targeted DNA Sequencing

DNA from PDO cells was extracted using the All Prep DNA/RNA Mini Kit (Qiagen, Germany). Library preparation was performed using a QIASeq™ custom targeted thirty gene DNA panel (Qiagen, Germany), detailed in **Supplementary Table 1**. Sequencing was performed using a MiSeq v2 (Illumina, USA), 300 cycle flow cell paired end at 500X coverage on a MiSeq™ (Illumina, USA) next generation sequencing (NGS) platform.

### RNA Sequencing

RNA from PDO cells was extracted using the AllPrep DNA/RNA Mini Kit. Ribosomal RNA (rRNA) was depleted using the rRNA depletion Kit (Human/Mouse/Rat) (New England BioLabs [NEB, USA]). Library preparation for total RNA sequencing was performed using NEBNext^®^ Ultra™ II RNA Library Preparation Kit for Illumina (NEB, USA). Sequencing was performed using a NextSeq HIGH 150 (Illumina, USA) flow cell on the NextSeq™ 500 (Illumina, USA) NGS platform.

### Single Cell RNA Sequencing

Irradiated (5 Gy/day over 5 days) and non-irradiated PDOs were dissociated into single cells after a 6-day recovery period. Libraries were prepared aiming for 7000 cell recovery, using 11 cycles of amplification of cDNA and 12 cycles for indexing PCR using the Chromium single cell 3’ kit (10X Genomics, USA). Sequencing was performed using a NextSeq™ 550 HIGH 150 flow cell and NGS platform.

### Genome Wide CRISPR-Cas9 Knockout

The GeCKO pooled library (#1000000048, Addgene, USA) was amplified as per the manufacturer’s instructions. Sufficient single guide RNA (sgRNA) coverage of the resulting library was confirmed on the Illumina MiSeq™. Lentivirus generation and subsequent transduction and selection of C80 and HT55 cell line was performed as described by Joung et al. (39). The CRC cell line used in the CRISPR-Cas9 experiments, C80 contained *APC*, *KRAS*, *SMAD4* and *TP53* mutations whereas the HT55 line contained *APC* and *TP53* mutations. Two cell populations were maintained, each with a coverage of 500 for each sgRNA (6.1X10^7^ cells). One population was treated with 5 Gy/day radiotherapy over 5 days and the second population remained untreated. Allowing for a 14-day, post-treatment period for cell recovery, 6.1x10^7 cells from each group were harvested, DNA extracted, and library preparation performed with individual barcoding of each group. NGS was performed using Illumina NextSeq™.

### *In Vitro* Cell Line and PDO Chemoradiotherapy Assays

HCT116 (1000 cells/well) and two radioresistant PDO lines (organoids equivalent to 5000 cells/well) were treated with 5-fluorouracil (5FU, PanReac-AppliChem, USA) or dual PI3K/mTOR inhibitors Apitolisib (GDC0980, Adooq Bioscience, USA) or Dactolisib (BEZ235, Selleckchem, USA), in 0.1% DMSO, with or without radiotherapy (5 Gy over 5 days for PDO lines and 1 Gy over 5 days for HCT 116). Ninety-six well assay plates were prepared. Drug treatment commenced on the third day after plating and radiotherapy on the fourth day. During the experiment, wells were replenished with 100μl of culture media (with or without drug) every 72 hours. The treatment ended on ninth day when all wells were replenished with 200μl of fresh culture media and incubated for a further 5 days to allow cell death or recovery. End-point viability was assessed with chemiluminescence using CellTiter-Glo 2.0 Assay (Promega, USA) and CellTiter-Glo^®^ 3D Cell Viability Assay for cell and PDO lines, respectively.

### Western Blots

The protein extracted from HCT116 cells following drug or radiotherapy treatment, was loaded at 20μg on to precast gels. Western blots were developed using protocols from Abcam Plc (UK) and primary antibodies Phospho-AKT Ser473 (4060S, Cell Signalling Technology, USA), AKT (9272S, Cell Signalling Technology, USA) and GAPDH D16H11 (5174S, Cell Signalling Technology, USA).

### Data Analysis

Prism V8 (Graphpad, USA) was used to generate dose-response graphs. Half maximal inhibitory concentrations (IC50) with 95% confidence interval (CI) were determined using linear regression. Two-way ANOVA mixed model analyses were performed using Prism V8 to assess significance in efficacy of radiotherapy or drug treatment on cell line and PDOs. Western blot experiments were quantified using ImageJ (https://imagej.nih.gov/ij/). A student t-test was performed using SPSS (IBM, USA) to assess significance of AKT phosphorylation or suppression. QIASeq data were analysed using the Biomedical Genomics Workbench (Qiagen, Germany). Mutation profile heatmap was generated using Oncoprint Chart. RNA NGS data were used to identify differentially expressed genes using pre-designed bioinformatic pipelines on Flow^®^ (Partek, UK). DESeq2 was applied to evaluate the differential gene expression during the transition of the two developmental stages using DEBrowser v1.16.1 (40, 41). Genes were considered differentially expressed when they had an adjusted p-value ≤ 0.01 (FDR); fold-change ≤ − 2. Differentially expressed genes were plotted using EnhancedVolcano (42). Over-representation analysis using gene sets from the MSigDB v7.4 (https://www.gsea-msigdb.org/gsea/index.jsp) was performed against differentially expressed genes using the R package clusterProfiler (43). The R packages were run on R Studio Desktop V1.3 (R Studio, USA) with R V4.0.2 (r-project.org, USA).

For single cell sequencing, read matrices were imported into R 4.0.4 (Lost Library Book) and BioConductor and the GRCh38 reference genome. Cells were filtered such that cells with > 5% mitochondrial reads or had < 200 features were excluded. Data were log normalised and non-linear dimensionality reduction performed *via* UMAP, and differential expression markers between clusters were found using the *FindMarkers* function of Seurat (44). Cell identity based on differential expression clusters was performed by a combination of KEGG pathway analysis and manual annotation based on the literature. Cell populations were segregated into those from irradiated organoids and those from control organoids and Scalable Bayesian Boolean Tensor Factorisation (SBBTF) using Python 3.7.3, which binarised gene expression with outcome (45).

Following the CRISPR/CAS9 screen, sequenced reads were analysed using the MaGECK pipeline (46). Briefly, sgRNA counts for each cell line were performed using the mageck *count* command and differential changes in sgRNA between cell types was carried out using the mageck *mle* command, using CNV data from the Depmap project to correct the sgRNA calls for alterations in CNV. Differential sgRNA data were processed using MaGECKFlute. Gene Ontology over-representation analysis was performed using the R package clusterProfiler. The ‘org.Hs.eg.db’ v3.13.052 genome-wide annotation was supplied for mapping Ensembl IDs to gene symbols. Significantly over-represented KEGG and gene oncogenic terms were defined from molecular signatures database v7.4 using differentially expressed genes filtered by *p* < 0.05. The ‘dotplot ()’ function was used to create visualisations of the top over-represented gene sets. Network modules of highly correlated genes was performed using the R package ‘WGCNA’ v1.70-353. The soft thresholding power was selected using the ‘pickSoftThreshold ()’ function and scale-free topology criterion. An adjacency matrix was created, containing the Pearson’s correlation coefficients of every pair of genes. The adjacency matrix was used to create a topological overlap matrix (TOM). The corresponding dissimilarity matrix (1 - TOM), was used for gene hierarchical clustering and subsequent module detection using the Dynamic Tree Cut algorithm. Each module detected was named using a colour.

## Data Availability Statement

RNA sequencing data is via Array Express at http://www.ebi.ac.uk/arrayexpress with the accession number E-MTAB-11849.

## Ethics Statement

The studies involving human participants were reviewed and approved by Project approval code 17-287, Human Biomaterials Resource Centre, Birmingham, United Kingdom; under ethical approval from Northwest - Haydock Research Ethics Committee (Reference: 15/NW/0079). The patients/participants provided their written informed consent to participate in this study.

## Author Contributions

KW and JB-S designed the experiments under the supervision of TI and AB. KW, OP, LT, AS, TS, and RH conducted the wet lab experiments and immunohistochemistry. LT conducted the CRISPR-Cas9 experiments. KW, OP, AS, LT, CB, JS, CW, RT, and TS were involved in the genomic work packages. KW, JB-S, ME, CY, and AB were responsible for data analysis including bioinformatics and data interpretation. KW and JBS wrote the manuscript with the support of ME, LT, JS, and AB. The remaining authors were also involved in the editing and final review of the manuscript. All authors contributed to the article and approved the submitted version.

## Funding

Cancer Research UK, Advanced Clinician Scientist Award (ref C31641/A23923).

## Conflict of Interest

The authors declare that the research was conducted in the absence of any commercial or financial relationships that could be construed as a potential conflict of interest.

## Publisher’s Note

All claims expressed in this article are solely those of the authors and do not necessarily represent those of their affiliated organizations, or those of the publisher, the editors and the reviewers. Any product that may be evaluated in this article, or claim that may be made by its manufacturer, is not guaranteed or endorsed by the publisher.
