# Supplementary material for: Patient Derived Organoids Confirm That PI3K/AKT Signalling Is an Escape Pathway for Radioresistance and a Target for Therapy in Rectal Cancer
Source: Front Oncol. 2022 Jul 4;12:920444. doi: 10.3389/fonc.2022.920444 (PMC9289101; doi:10.3389/fonc.2022.920444)
Supplement: Supplementary file 1 [file DataSheet_1.docx]

**Supplementary Table 1: Genes tested using the** **QIASeq™ (Qiagen, Germany) custom targeted DNA sequencing panel**

| Gene | bp ROI | bp not covered by fragments <= 150 bp | bp not covered by fragments <= 250 bp |
| --- | --- | --- | --- |
| MSH6 | 4183 | 0 | 0 |
| BRAF | 2660 | 0 | 0 |
| TCF7L2 | 2425 | 0 | 0 |
| BCL9L | 4580 | 0 | 0 |
| TP53 | 1383 | 0 | 0 |
| B2M | 413 | 0 | 0 |
| TGIF1 | 1365 | 0 | 0 |
| NRAS | 610 | 0 | 0 |
| PIK3CA | 3407 | 0 | 0 |
| GNAS | 4186 | 0 | 0 |
| SMAD4 | 1769 | 0 | 0 |
| BMPR2 | 3247 | 0 | 0 |
| PTEN | 1302 | 0 | 0 |
| RPL22 | 619 | 0 | 0 |
| SMAD2 | 1504 | 0 | 0 |
| ATM | 9791 | 0 | 0 |
| POLE | 7351 | 0 | 0 |
| ARID1A | 7058 | 0 | 0 |
| FBXW7 | 2758 | 0 | 0 |
| RNF43 | 2442 | 0 | 0 |
| MLH1 | 2461 | 0 | 0 |
| MSH2 | 3107 | 0 | 0 |
| KRAS | 737 | 0 | 0 |
| ELF3 | 1256 | 0 | 0 |
| POLD1 | 3662 | 0 | 0 |
| CTNNB1 | 2486 | 0 | 0 |
| ZFP36L2 | 1505 | 0 | 0 |
| APC | 8857 | 0 | 0 |
| SOX9 | 1560 | 0 | 0 |
| ACVR2A | 1652 | 0 | 0 |

track name='QIAseq_DNA_panel.CDHS-14542Z-1197.roi' description='QIAseq_DNA_panel.CDHS-14542Z-1197.roi'

chr1 6246726 6246881 RPL22

chr1 6252984 6253119 RPL22

chr1 6257706 6257821 RPL22

chr1 6259424 6259638 RPL22

chr1 27022889 27024036 ARID1A

chr1 27056136 27056359 ARID1A

chr1 27057637 27058100 ARID1A

chr1 27059161 27059288 ARID1A

chr1 27087341 27087592 ARID1A

chr1 27087869 27087969 ARID1A

chr1 27088637 27088815 ARID1A

chr1 27089458 27089781 ARID1A

chr1 27092706 27092862 ARID1A

chr1 27092942 27093062 ARID1A

chr1 27094275 27094495 ARID1A

chr1 27097604 27097822 ARID1A

chr1 27098985 27099128 ARID1A

chr1 27099297 27099483 ARID1A

chr1 27099831 27099992 ARID1A

chr1 27100065 27100213 ARID1A

chr1 27100287 27100394 ARID1A

chr1 27100814 27101716 ARID1A

chr1 27102062 27102203 ARID1A

chr1 27105508 27107252 ARID1A

chr1 115251150 115251280 NRAS

chr1 115252184 115252354 NRAS

chr1 115256415 115256604 NRAS

chr1 115258665 115258786 NRAS

chr1 201980259 201980432 ELF3

chr1 201981079 201981311 ELF3

chr1 201981466 201981569 ELF3

chr1 201981762 201981892 ELF3

chr1 201981973 201982033 ELF3

chr1 201982069 201982169 ELF3

chr1 201982304 201982431 ELF3

chr1 201982951 201983157 ELF3

chr1 201984331 201984456 ELF3

chr10 89624221 89624310 PTEN

chr10 89653776 89653871 PTEN

chr10 89685264 89685319 PTEN

chr10 89690797 89690851 PTEN

chr10 89692764 89693013 PTEN

chr10 89711869 89712021 PTEN

chr10 89717604 89717781 PTEN

chr10 89720645 89720880 PTEN

chr10 89725038 89725234 PTEN

chr10 114710510 114710709 TCF7L2

chr10 114710960 114711037 TCF7L2

chr10 114711236 114711371 TCF7L2

chr10 114724309 114724388 TCF7L2

chr10 114799778 114799890 TCF7L2

chr10 114849150 114849304 TCF7L2

chr10 114886632 114886645 TCF7L2

chr10 114889618 114889751 TCF7L2

chr10 114900937 114901080 TCF7L2

chr10 114903676 114903789 TCF7L2

chr10 114905764 114905861 TCF7L2

chr10 114910736 114910887 TCF7L2

chr10 114911478 114911648 TCF7L2

chr10 114912086 114912204 TCF7L2

chr10 114917774 114917833 TCF7L2

chr10 114918420 114918481 TCF7L2

chr10 114919673 114919756 TCF7L2

chr10 114920372 114920455 TCF7L2

chr10 114921332 114921349 TCF7L2

chr10 114925308 114925736 TCF7L2

chr11 108098346 108098428 ATM

chr11 108098497 108098620 ATM

chr11 108099899 108100055 ATM

chr11 108106391 108106566 ATM

chr11 108114674 108114850 ATM

chr11 108115509 108115758 ATM

chr11 108117685 108117859 ATM

chr11 108119654 108119834 ATM

chr11 108121422 108121804 ATM

chr11 108122558 108122763 ATM

chr11 108123538 108123644 ATM

chr11 108124535 108124771 ATM

chr11 108126936 108127072 ATM

chr11 108128202 108128338 ATM

chr11 108129707 108129807 ATM

chr11 108137892 108138074 ATM

chr11 108139131 108139341 ATM

chr11 108141785 108141878 ATM

chr11 108141972 108142138 ATM

chr11 108143253 108143339 ATM

chr11 108143443 108143584 ATM

chr11 108150212 108150340 ATM

chr11 108151716 108151900 ATM

chr11 108153431 108153611 ATM

chr11 108154948 108155205 ATM

chr11 108158321 108158447 ATM

chr11 108159698 108159835 ATM

chr11 108160323 108160533 ATM

chr11 108163340 108163525 ATM

chr11 108164034 108164209 ATM

chr11 108165648 108165791 ATM

chr11 108168008 108168114 ATM

chr11 108170435 108170617 ATM

chr11 108172369 108172521 ATM

chr11 108173574 108173761 ATM

chr11 108175396 108175584 ATM

chr11 108178618 108178716 ATM

chr11 108180881 108181047 ATM

chr11 108183132 108183230 ATM

chr11 108186544 108186643 ATM

chr11 108186732 108186845 ATM

chr11 108188094 108188253 ATM

chr11 108190675 108190790 ATM

chr11 108192022 108192152 ATM

chr11 108196031 108196276 ATM

chr11 108196779 108196957 ATM

chr11 108198366 108198490 ATM

chr11 108199742 108199970 ATM

chr11 108200935 108201153 ATM

chr11 108202165 108202289 ATM

chr11 108202600 108202769 ATM

chr11 108203483 108203632 ATM

chr11 108204607 108204700 ATM

chr11 108205690 108205841 ATM

chr11 108206566 108206693 ATM

chr11 108213943 108214103 ATM

chr11 108216464 108216640 ATM

chr11 108218000 108218097 ATM

chr11 108224487 108224612 ATM

chr11 108225532 108225606 ATM

chr11 108235803 108235950 ATM

chr11 108236046 108236240 ATM

chr11 118769118 118770222 BCL9L

chr11 118770620 118770912 BCL9L

chr11 118771322 118773622 BCL9L

chr11 118773693 118773788 BCL9L

chr11 118773939 118774166 BCL9L

chr11 118778186 118778316 BCL9L

chr11 118778973 118779369 BCL9L

chr11 118780617 118780653 BCL9L

chr12 25362723 25362850 KRAS

chr12 25368369 25368499 KRAS

chr12 25378542 25378712 KRAS

chr12 25380162 25380351 KRAS

chr12 25398202 25398323 KRAS

chr12 133201277 133201401 POLE

chr12 133201485 133201585 POLE

chr12 133202225 133202361 POLE

chr12 133202697 133202908 POLE

chr12 133208895 133209099 POLE

chr12 133209244 133209386 POLE

chr12 133210766 133210969 POLE

chr12 133212472 133212615 POLE

chr12 133214594 133214730 POLE

chr12 133215705 133215889 POLE

chr12 133218227 133218442 POLE

chr12 133218757 133218988 POLE

chr12 133219086 133219320 POLE

chr12 133219400 133219587 POLE

chr12 133219804 133219921 POLE

chr12 133219987 133220151 POLE

chr12 133220417 133220568 POLE

chr12 133225509 133225663 POLE

chr12 133225886 133226106 POLE

chr12 133226257 133226480 POLE

chr12 133233716 133233849 POLE

chr12 133233929 133234020 POLE

chr12 133234448 133234561 POLE

chr12 133235875 133236100 POLE

chr12 133237549 133237755 POLE

chr12 133238107 133238275 POLE

chr12 133240584 133240739 POLE

chr12 133240950 133241053 POLE

chr12 133241882 133242041 POLE

chr12 133244083 133244239 POLE

chr12 133244936 133245093 POLE

chr12 133245215 133245328 POLE

chr12 133245391 133245530 POLE

chr12 133248795 133248913 POLE

chr12 133249207 133249430 POLE

chr12 133249744 133249868 POLE

chr12 133250155 133250298 POLE

chr12 133251978 133252108 POLE

chr12 133252315 133252411 POLE

chr12 133252674 133252795 POLE

chr12 133253126 133253244 POLE

chr12 133253943 133254034 POLE

chr12 133254158 133254310 POLE

chr12 133256077 133256242 POLE

chr12 133256534 133256637 POLE

chr12 133256758 133256813 POLE

chr12 133257187 133257278 POLE

chr12 133257718 133257870 POLE

chr12 133263834 133263906 POLE

chr15 45003739 45003816 B2M

chr15 45007615 45007927 B2M

chr15 45008521 45008545 B2M

chr17 7572921 7573013 TP53

chr17 7573921 7574038 TP53

chr17 7576531 7576589 TP53

chr17 7576619 7576662 TP53

chr17 7576847 7576931 TP53

chr17 7577013 7577160 TP53

chr17 7577493 7577613 TP53

chr17 7578171 7578294 TP53

chr17 7578365 7578559 TP53

chr17 7579306 7579595 TP53

chr17 7579694 7579726 TP53

chr17 7579833 7579917 TP53

chr17 56432298 56432352 RNF43

chr17 56434823 56436189 RNF43

chr17 56437504 56437617 RNF43

chr17 56438138 56438310 RNF43

chr17 56439899 56440014 RNF43

chr17 56440630 56440772 RNF43

chr17 56440881 56440966 RNF43

chr17 56448266 56448399 RNF43

chr17 56492681 56492943 RNF43

chr17 70117527 70117968 SOX9

chr17 70118854 70119118 SOX9

chr17 70119678 70120533 SOX9

chr18 3447732 3447800 TGIF1

chr18 3449624 3449659 TGIF1

chr18 3450482 3450508 TGIF1

chr18 3451972 3452385 TGIF1

chr18 3456346 3456583 TGIF1

chr18 3457357 3457943 TGIF1

chr18 45368192 45368326 SMAD2

chr18 45371705 45371860 SMAD2

chr18 45372028 45372176 SMAD2

chr18 45374840 45375063 SMAD2

chr18 45377639 45377703 SMAD2

chr18 45391424 45391509 SMAD2

chr18 45394688 45394833 SMAD2

chr18 45395608 45395812 SMAD2

chr18 45396840 45396940 SMAD2

chr18 45422886 45423132 SMAD2

chr18 48573411 48573670 SMAD4

chr18 48575050 48575235 SMAD4

chr18 48575659 48575699 SMAD4

chr18 48581145 48581368 SMAD4

chr18 48584489 48584619 SMAD4

chr18 48584704 48584831 SMAD4

chr18 48586230 48586291 SMAD4

chr18 48591787 48591981 SMAD4

chr18 48593383 48593562 SMAD4

chr18 48603002 48603151 SMAD4

chr18 48604620 48604842 SMAD4

chr19 50902103 50902315 POLD1

chr19 50902622 50902746 POLD1

chr19 50905029 50905186 POLD1

chr19 50905250 50905386 POLD1

chr19 50905456 50905635 POLD1

chr19 50905705 50905797 POLD1

chr19 50905863 50906003 POLD1

chr19 50906304 50906481 POLD1

chr19 50906744 50906859 POLD1

chr19 50909433 50909584 POLD1

chr19 50909658 50909779 POLD1

chr19 50910234 50910436 POLD1

chr19 50910578 50910677 POLD1

chr19 50911958 50912163 POLD1

chr19 50912373 50912497 POLD1

chr19 50912770 50912928 POLD1

chr19 50916677 50916783 POLD1

chr19 50916993 50917141 POLD1

chr19 50918066 50918252 POLD1

chr19 50918689 50918852 POLD1

chr19 50918975 50919088 POLD1

chr19 50919647 50919790 POLD1

chr19 50919861 50919985 POLD1

chr19 50920296 50920359 POLD1

chr19 50920423 50920531 POLD1

chr19 50921093 50921209 POLD1

chr2 43451452 43452896 ZFP36L2

chr2 43453398 43453459 ZFP36L2

chr2 47630325 47630546 MSH2

chr2 47635534 47635699 MSH2

chr2 47637227 47637516 MSH2

chr2 47639547 47639704 MSH2

chr2 47641402 47641562 MSH2

chr2 47643429 47643573 MSH2

chr2 47656875 47657085 MSH2

chr2 47672681 47672801 MSH2

chr2 47690164 47690298 MSH2

chr2 47693791 47693952 MSH2

chr2 47698098 47698206 MSH2

chr2 47702158 47702414 MSH2

chr2 47703500 47703715 MSH2

chr2 47705405 47705663 MSH2

chr2 47707829 47708015 MSH2

chr2 47709912 47710093 MSH2

chr2 47739436 47739578 MSH2

chr2 48010367 48010637 MSH6

chr2 48018060 48018267 MSH6

chr2 48023027 48023207 MSH6

chr2 48025744 48028299 MSH6

chr2 48030553 48030829 MSH6

chr2 48032043 48032171 MSH6

chr2 48032751 48032851 MSH6

chr2 48033337 48033502 MSH6

chr2 48033585 48033795 MSH6

chr2 48033912 48034004 MSH6

chr2 148602716 148602781 ACVR2A

chr2 148653864 148654082 ACVR2A

chr2 148657021 148657141 ACVR2A

chr2 148657307 148657472 ACVR2A

chr2 148672754 148672908 ACVR2A

chr2 148674846 148675000 ACVR2A

chr2 148676010 148676166 ACVR2A

chr2 148677793 148677918 ACVR2A

chr2 148680536 148680685 ACVR2A

chr2 148683594 148683735 ACVR2A

chr2 148684643 148684848 ACVR2A

chr2 203242192 203242278 BMPR2

chr2 203329526 203329707 BMPR2

chr2 203332236 203332417 BMPR2

chr2 203378436 203378557 BMPR2

chr2 203379605 203379707 BMPR2

chr2 203383539 203383780 BMPR2

chr2 203384804 203384929 BMPR2

chr2 203395511 203395682 BMPR2

chr2 203397302 203397460 BMPR2

chr2 203407028 203407175 BMPR2

chr2 203417433 203417616 BMPR2

chr2 203419969 203421259 BMPR2

chr2 203424413 203424674 BMPR2

chr20 57415156 57415946 GNAS

chr20 57428315 57430393 GNAS

chr20 57466776 57466925 GNAS

chr20 57470661 57470744 GNAS

chr20 57473990 57474045 GNAS

chr20 57478577 57478645 GNAS

chr20 57478721 57478851 GNAS

chr20 57480432 57480540 GNAS

chr20 57484211 57484276 GNAS

chr20 57484399 57484483 GNAS

chr20 57484570 57484639 GNAS

chr20 57484733 57484864 GNAS

chr20 57485000 57485141 GNAS

chr20 57485383 57485461 GNAS

chr20 57485732 57485889 GNAS

chr3 37035033 37035159 MLH1

chr3 37038104 37038205 MLH1

chr3 37042440 37042549 MLH1

chr3 37045886 37045970 MLH1

chr3 37048476 37048559 MLH1

chr3 37050299 37050401 MLH1

chr3 37053305 37053358 MLH1

chr3 37053496 37053595 MLH1

chr3 37055917 37056040 MLH1

chr3 37058991 37059095 MLH1

chr3 37061795 37061959 MLH1

chr3 37067122 37067503 MLH1

chr3 37070269 37070428 MLH1

chr3 37081671 37081790 MLH1

chr3 37083753 37083827 MLH1

chr3 37089004 37089179 MLH1

chr3 37090002 37090105 MLH1

chr3 37090389 37090513 MLH1

chr3 37091971 37092149 MLH1

chr3 41265554 41265577 CTNNB1

chr3 41266011 41266249 CTNNB1

chr3 41266439 41266703 CTNNB1

chr3 41266819 41267068 CTNNB1

chr3 41267145 41267357 CTNNB1

chr3 41268693 41268848 CTNNB1

chr3 41274826 41274940 CTNNB1

chr3 41275014 41275363 CTNNB1

chr3 41275624 41275793 CTNNB1

chr3 41277209 41277339 CTNNB1

chr3 41277834 41277995 CTNNB1

chr3 41278073 41278205 CTNNB1

chr3 41279501 41279572 CTNNB1

chr3 41280619 41280838 CTNNB1

chr3 178916608 178916970 PIK3CA

chr3 178917472 178917692 PIK3CA

chr3 178919072 178919333 PIK3CA

chr3 178921326 178921582 PIK3CA

chr3 178922285 178922381 PIK3CA

chr3 178927377 178927493 PIK3CA

chr3 178927968 178928131 PIK3CA

chr3 178928213 178928358 PIK3CA

chr3 178935992 178936127 PIK3CA

chr3 178936978 178937070 PIK3CA

chr3 178937353 178937528 PIK3CA

chr3 178937731 178937845 PIK3CA

chr3 178938768 178938950 PIK3CA

chr3 178941863 178941980 PIK3CA

chr3 178942482 178942614 PIK3CA

chr3 178943744 178943833 PIK3CA

chr3 178947054 178947235 PIK3CA

chr3 178947786 178947914 PIK3CA

chr3 178948007 178948169 PIK3CA

chr3 178951876 178952157 PIK3CA

chr4 153244027 153244306 FBXW7

chr4 153245330 153245551 FBXW7

chr4 153247152 153247388 FBXW7

chr4 153249354 153249546 FBXW7

chr4 153250818 153250942 FBXW7

chr4 153251878 153252025 FBXW7

chr4 153253742 153253876 FBXW7

chr4 153258948 153259093 FBXW7

chr4 153268076 153268228 FBXW7

chr4 153269820 153269886 FBXW7

chr4 153271188 153271281 FBXW7

chr4 153273616 153273887 FBXW7

chr4 153303335 153303492 FBXW7

chr4 153332419 153332960 FBXW7

chr5 112043409 112043584 APC

chr5 112090582 112090727 APC

chr5 112102017 112102112 APC

chr5 112102880 112103092 APC

chr5 112111320 112111439 APC

chr5 112116481 112116605 APC

chr5 112128137 112128231 APC

chr5 112136970 112137085 APC

chr5 112151186 112151295 APC

chr5 112154657 112155046 APC

chr5 112157587 112157693 APC

chr5 112162799 112162949 APC

chr5 112163620 112163708 APC

chr5 112164547 112164674 APC

chr5 112170642 112170867 APC

chr5 112173244 112179828 APC

chr7 140415822 140415841 BRAF

chr7 140426161 140426321 BRAF

chr7 140434391 140434575 BRAF

chr7 140439606 140439751 BRAF

chr7 140449081 140449223 BRAF

chr7 140453069 140453198 BRAF

chr7 140453981 140454038 BRAF

chr7 140476706 140476893 BRAF

chr7 140477785 140477880 BRAF

chr7 140481370 140481498 BRAF

chr7 140482815 140482962 BRAF

chr7 140487342 140487389 BRAF

chr7 140494102 140494272 BRAF

chr7 140500156 140500286 BRAF

chr7 140501206 140501365 BRAF

chr7 140507754 140507867 BRAF

chr7 140508686 140508800 BRAF

chr7 140534403 140534677 BRAF

chr7 140549905 140550017 BRAF

chr7 140624360 140624508 BRAF

**Supplementary Table 2: PDO mutations identified using the QIASeq™ (Qiagen, Germany) custom targeted DNA sequencing panel**

|  | **PDO 411** | **PDO 884** | **PDO 389** | **PDO 653** | **PDO 064** | **PDO 557** |
| --- | --- | --- | --- | --- | --- | --- |
| *APC* | stop gained p. Gln1378* | stop gained p. Tyr1376* | frameshift p. Gln1338fs | frameshift p. Val1479fs | stop gained p. Arg876* | missense p. Ala921Ser |
| *TP53* | missense p. Arg248Gln  R248Q | missense p. Arg175His R175H | missense p. Gly245Asp G245D | in frame deletion p. Pro191del P191del | missense p. Arg282Trp R282W | - |
| *MSH6* | frameshift p. Leu1330fs L1330fs | frameshift p. Phe1088fs F1088fs | - | - | - | frameshift p. Phe1104fs F1104fs |
| *SOX9* | - | frameshift p. Tyr297fs Y297fs | frameshift p. Ser323fs S323fs | stop gained p. Gln1378* Q1378* | - | - |
| *TCF7L2* | frameshift p. Lys485fs | - | frameshift p. Lys485fs | - | - | - |
| *PIK3CA* | missense p. Glu545Lys E545K | - | - | - | - | missense p. Arg88Gln R88Q |
| *GNAS* | missense p. Thr225Pro | missense p. Thr225Pro | - | - | - | - |
| *SMAD4* | - | - | missense p. Arg496His | frameshift p. Trp302fs | - | - |
| *BMPR2* | stop gained p. Arg147* | - | - | - | - | frameshift p. Asn583fs |
| *FBXW7* | - | - | - | missense p. Arg465His R465H | missense p. Ser582Leu S582L | - |
| *MSH2* | missense p. Asp167Val | - | frameshift p. Leu458fs L458fs | - | - | - |
| *KRAS* | - | - | - | missense p. Gly12Asp G12D | - | missense p. Gly13Arg G13R |
| *BRAF* | missense p. Val600Glu | - | - | - | - | - |
| *BCL9L* | - | - | - | - | - | frameshift p. Pro449fs |
| *TGIF1* | - | - | - | - | frameshift p. Phe337fs | - |
| *NRAS* | - | missense p. Gly13Arg | - | - | - | - |
| *PTEN* | - | - | - | - | - | missense p. Cys136Tyr |
| *RPL22* | - | - | - | - | - | frameshift p. Lys15fs |
| *POLE* | frameshift p. Val1446fs | - | - | - | - | - |
| *ACVR2A* | - | - | - | - | - | frameshift p. Lys437fs |

|  | PCK | CDX2 |
| --- | --- | --- |
| 064 | 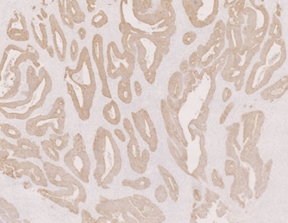 | 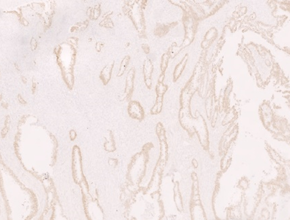 |
| 884 | 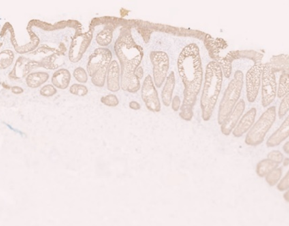 | 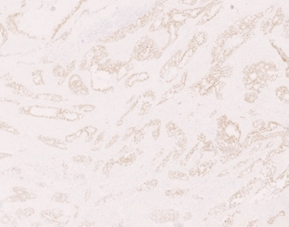 |
| 389 | 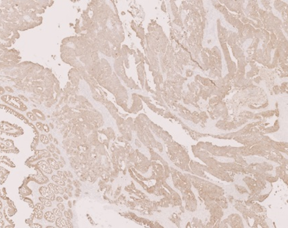 | 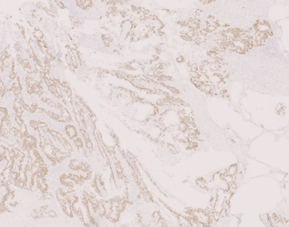 |
| 411 | 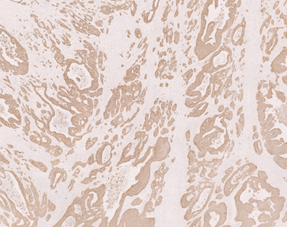 | 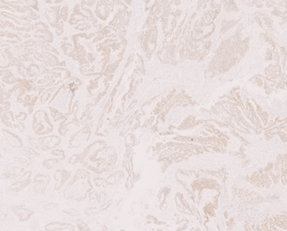 |
|  |  |  |
|  |  |  |

**Supplementary Figure 1: Immunohistochemistry of parent tumour tissue from which PDO lines were derived from**

*PCK – pancytokeratin*
